# Key issues in recruitment to randomised controlled trials with very different interventions: a qualitative investigation of recruitment to the SPARE trial (CRUK/07/011)

**DOI:** 10.1186/1745-6215-12-78

**Published:** 2011-03-15

**Authors:** Sangeetha Paramasivan, Robert Huddart, Emma Hall, Rebecca Lewis, Alison Birtle, Jenny L Donovan

**Affiliations:** 1 School of Social and Community Medicine, University of Bristol, 39 Canynge Hall, Whatley Road, Bristol BS8 2PS, UK; 2 Section of Academic Radiotherapy, The Institute of Cancer Research, Orchard House, The Royal Marsden Hospital, Sutton SM2 5PT, UK; 3 The ICR Clinical Trials & Statistics Unit (ICR-CTSU), The Institute of Cancer Research, Sir Richard Doll Building, Cotswold Road, Sutton SM2 5NG, UK; 4 Rosemere Cancer Centre, Lancashire Teaching Hospitals NHS Foundation Trust, Royal Preston Hospital, Sharoe Green Lane North, Preston PR2 9HT, UK; 5 School of Cancer and Imaging Science, Faculty of Medical and Human Sciences, University of Manchester, Stopford Building, Oxford Road, Manchester M13 9PT, UK

## Abstract

**Background:**

Recruitment to randomised controlled trials (RCTs) with very different treatment arms is often difficult. The ProtecT (Prostate testing for cancer and Treatment) study successfully used qualitative research methods to improve recruitment and these methods were replicated in five other RCTs facing recruitment difficulties. A similar qualitative recruitment investigation was undertaken in the SPARE (Selective bladder Preservation Against Radical Excision) feasibility study to explore reasons for low recruitment and attempt to improve recruitment rates by implementing changes suggested by qualitative findings.

**Methods:**

In Phase I of the investigation, reasons for low levels of recruitment were explored through content analysis of RCT documents, thematic analysis of interviews with trial staff and recruiters, and conversation analysis of audio-recordings of recruitment appointments. Findings were presented to the trial management group and a plan of action was agreed. In Phase II, changes to design and conduct were implemented, with training and feedback provided for recruitment staff.

**Results:**

Five key challenges to trial recruitment were identified in Phase I: (a) Investigators and recruiters had considerable difficulty articulating the trial design in simple terms; (b) The recruitment pathway was complicated, involving staff across different specialties/centres and communication often broke down; (c) Recruiters inadvertently used 'loaded' terminology such as 'gold standard' in study information, leading to unbalanced presentation; (d) Fewer eligible patients were identified than had been anticipated; (e) Strong treatment preferences were expressed by potential participants and trial staff in some centres. In Phase II, study information (patient information sheet and flowchart) was simplified, the recruitment pathway was focused around lead recruiters, and training sessions and 'tips' were provided for recruiters. Issues of patient eligibility were insurmountable, however, and the independent Trial Steering Committee advised closure of the SPARE trial in February 2010.

**Conclusions:**

The qualitative investigation identified the key aspects of trial design and conduct that were hindering recruitment, and a plan of action that was acceptable to trial investigators and recruiters was implemented. Qualitative investigations can thus be used to elucidate challenges to recruitment in trials with very different treatment arms, but require sufficient time to be undertaken successfully.

**Trial Registration:**

CRUK/07/011; ISRCTN61126465

## Background

Randomised controlled trials (RCTs) often encounter recruitment difficulties, even when the interventions appear relatively uncontroversial [1]. RCTs including a surgical arm face particular challenges [2,3]. A major challenge is that unless surgical procedures are being compared with each other, the comparison arm is likely to be very different [4]. Considering randomisation between surgery and another treatment such as radiotherapy, chemotherapy or some form of delayed intervention or monitoring is often difficult for clinicians and patients. Clinicians have to come to terms with the fact that there is uncertainty/equipoise about treatments they may have been recommending for some time and accept that the treatments have not been robustly evaluated and so may not be the most effective or cost-effective. Participating in the RCT is likely to mean collaboration and coordination with colleagues in other specialties where the culture may be somewhat different. For patients, the idea that there is uncertainty over the comparative effectiveness of such different interventions - that such different treatments may lead to similar long term outcomes, particularly when short-term side effects are very different - can be very difficult to accept. When the disease being treated is cancer, with strong lay beliefs and connotations of 'life and death', RCTs involving a comparison of surgical procedures with other techniques are fraught with difficulties.

However, in many areas of cancer treatment/therapy, there is uncertainty over the most effective treatment, and often the most controversial questions focus in areas where the alternatives may include a surgical option. For example, in muscle invasive bladder cancer, most research has focussed on individual aspects of the two main modes of treatment: radical surgery (cystectomy - removal of the bladder) or conservative treatment based on radiotherapy (either alone or as part of a programme of selective-bladder preservation [SBP] where radiotherapy is used in those with good response to neo-adjuvant chemotherapy and cystectomy is performed in poor responders). Case series (with all their attendant flaws) have demonstrated similar survival outcomes for contemporary cystectomy, radiotherapy and SBP approaches, but the need for rigorous evidence in the form of a randomised phase III trial to demonstrate that these approaches are comparable has recently been very strongly encouraged [5].

The SPARE (Selective bladder Preservation Against Radical Excision) trial (CRUK/07/011; ISRCTN61126465) was set up to compare radical surgery with SBP in patients with muscle invasive transitional cell carcinoma of the bladder. The primary objective was to demonstrate that SBP was not-inferior to surgery in terms of overall survival and this required a sample size of 1015 patients. It was anticipated that this would be a difficult trial because of the comparison between surgery and radiotherapy, and so a study to investigate the feasibility of recruitment began in July 2007 and included a qualitative study of patient perceptions and experiences [6]. Recruitment proceeded slowly and failed to meet original feasibility targets. In April 2009, with the support of the independent Trial Steering Committee (TSC), the Trial Management Group's (TMG) request to the Funder to extend the feasibility study by one year was approved and a target accrual rate of at least 6 patients per month was set, to be achieved prior to completion of the three year feasibility phase. At that point, 34 patients (out of a target 110) had been recruited over a period of 22 months. A focussed qualitative study of recruitment issues was initiated by the Chief Investigator and the TMG to try to identify any difficulties that might be amenable to improvement, albeit within a very tight timescale. This recruitment investigation built on findings from similar studies in other RCTs [7-9] and was based on the application of an adaptation of a complex intervention to improve recruitment [10]. This paper reports on the findings of this qualitative recruitment investigation.

## Methods

The qualitative recruitment investigation comprised two iterative and overlapping phases of data collection, data analysis, feedback and training (Table 1). The design was based broadly on the ProtecT (Prostate testing for cancer and Treatment) complex recruitment intervention [10], but with adaptations suggested by the results from similar applications of qualitative research to improve recruitment in five other RCTs [7]. The two-stage design of the recruitment investigation in the SPARE RCT comprised a first phase of intensive data collection and analysis to investigate the issues that were leading to low levels of recruitment and an 'intervention' phase to improve recruitment. Detailed research methods are described below.

**Table 1:** SPARE trial qualitative recruitment investigation - design and progression

	Time period			
	
	Months 1-3	Month 4	Months 5-7	Months 8-10
**Phase 1 **Data collection and analysis	Interviews with main trial team including oncologists; documentary analysis of protocol, some literature and Patient Information Sheet; investigation of screening logs		Interviews with clinicians, including urologists, and recruiters (outside main trial team)	
	
	Audio-recording of recruitment appointments			

**Phase 2 **Feedback and training		Feedback to main trial team and agreed joint plan of action	Changes developed and implemented	
	
			Recruitment tips and training sessions	

The qualitative recruitment investigation comprised two iterative and

overlapping phases - a first phase of intensive data collection and analysis to

investigate the issues that were leading to low levels of recruitment and an

'intervention' phase to improve recruitment through feedback and training.

The investigation carried out over 10 months is represented in Table 1.

### Phase I

Ethical approval was gained from the South East Research Ethics Committee (Reference number: 06/MRE01/95) to conduct the recruitment investigation as an integrated part of the SPARE feasibility study, as recommended by de Salis et al. [7]. Data collection and analysis began immediately, including:

#### 1. Document reviews

The recruitment investigation team (SP and JLD) interrogated the SPARE trial protocol and examined the SPARE trial Patient Information Sheet (PIS) to investigate whether any aspects were unclear or potentially open to misinterpretation. A rapid literature search identified a recent review in the Lancet summarising current evidence [5]. Content analysis was used to summarise aspects of the trial protocol concerned with eligibility and recruitment, and identify any potentially misleading sections in the PIS [11-13].

#### 2. In-depth interviews

Two interview topic guides were developed, based on those used in previous studies [7], one for the main SPARE TMG team closely involved in the design and coordination of the trial: chief investigator, scientific lead, a major clinical contributor, and the trial manager; and another for those who directly recruited patients including clinicians, research fellows and research nurses. All interviews were conducted by SP after obtaining informed consent. Interviewees were asked about the background and purpose of the trial; their role in the trial; recruitment training they had received; the SPARE recruitment pathway, including its processes and any difficulties; structural and organisational issues; and their views about the trial treatments and randomisation, the importance of the trial and how to improve recruitment. In addition, interviewees who were recruiters were also asked about how they introduced the trial and explained randomisation in appointments already conducted, recruitment strategies used, acceptability of the trial design and their satisfaction with the adequacy of the discussion and outcome of the appointments. Interviews were conducted purposively - first with members of the SPARE TMG, then clinicians/nurses involved in recruitment, and then with recruiters in centres not involved in the SPARE TMG.

Thematic analysis, using techniques of constant comparison, was undertaken by SP on transcripts of in-depth interviews. This involved comparing emerging themes and codes within transcripts and across the dataset [14,15]. The coding carried out using ATLAS-ti qualitative data analysis software was cross-checked by JLD and inconsistencies resolved. Descriptive accounts were produced by SP and discussed with JLD.

#### 3. Audio recording of discussions between potential RCT participants and recruitment staff

Audio recording of recruitment appointments had been shown to be extremely important for understanding key issues in the ProtecT study [16], but was found to be very difficult to operationalise in other RCTs [7]. Eight digital recorders were delivered to seven centres participating in SPARE with documents explaining the ethical requirements and instructions for use, and strong encouragement was given by the TMG to record appointments. Data analysis included thematic methods (as described above for interviews) and targeted conversation analysis to identify problematic aspects of interactions [16].

#### 4. Details collected in SPARE trial screening logs

Clinical centres open for recruitment to SPARE were asked to keep detailed logs of patients who were investigated for trial eligibility to record what happened to them, including reasons for including/not including them in recruitment and any reasons given for declining involvement. They were also asked to document the recruitment pathways of patients, beginning from the diagnosis of bladder cancer to making a decision about participating in the SPARE trial. Simple counts, cross tabulations and content analysis were combined to summarise the data [12] and describe the recruitment pathways.

### Phase II

This phase commenced with a meeting between the recruitment investigation team and leading members of the SPARE TMG, including patient representatives, at which a summary of anonymised findings from Phase I were presented by JLD. The findings are detailed in the results, below, and were used to draw up a plan of action for Phase II which was agreed between the SPARE TMG and recruitment investigation team and comprised:

1. A new version of the PIS including a simpler version of the study flowchart.

2. A programme of activity to try to increase recruitment, including:

a. A document of 'tips for recruitment' based on the one used in the ProtecT complex intervention, but adapted specifically for SPARE based on the findings of the recruitment study.

b. Dedicated training sessions for clinical centre staff working on SPARE.

The impact of Phase II was evaluated observationally through simple counts of recruited participants and with qualitative (thematic) analysis of comments made by clinicians and recruiters in SPARE who had read the 'tips' document and/or attended the training sessions.

## Results

The Phase I data collected from the in-depth interviews, documents, tape recordings and screening logs were analysed first. The content analysis of the trial protocol, PIS and recent review publication [5] provided important background information. The first set of interviews comprised seven participants, six of whom were members of the main trial team, and included three oncologists. Four participants, including two urologists (surgeons) were then recruited from non-TMG centres. In total nine recruiters and two non-recruiters were interviewed across four centres. Although clear instructions were provided and the recruiters took away the recorders enthusiastically, only four audio-recordings of recruitment appointments were made. The reasons for the limited number of recordings were not expressed clearly in this study, but were likely to be similar to those found elsewhere [7], with additional difficulty in SPARE because of the complicated and prolonged recruitment period (see below). Simple counts and content analysis of screening logs revealed that numbers of eligible patients varied considerably between recruitment centres, partly due to the variations in how systematically the screening logs were maintained in each centre. Less than half (43.4%) of patients screened were deemed potentially eligible for the SPARE trial, and less than a half (43.3%) of those were actually approached to discuss the trial (Table 2). 'Treatment preferences' were the most commonly reported reasons for refusal to be randomised (73.7%). In most centres, patients appeared to favour (or disfavour) one arm in particular, and overall there was an indication of greater preference for bladder preservation (58.9%) than for surgery (30.4%).

**Table 2:** SPARE trial screening log summary information

Total no. of patients considered for SPARE (22 centres; 22 months)	585
Ineligible	331 (56.6%)

Potentially eligible	254 (43.4%)
A. Approached	110 (43.3%)
1. Recruited	34 (30.9%)
2. Declined	76 (69.1%)
Due to preference	56 (73.7%)
*Surgery*	*17 (30.4%)*
*Radiotherapy/No surgery*	*33 (58.9%)*
*No chemo*	*6 (10.7%)*
Unknown/Other	20 (26.3%)

B. Not approached	105 (41.3%)
1. Due to preference	28 (26.7%)
*Surgery*	*11 (39.3%)*
*Radiotherapy/No surgery*	*10 (35.7%)*
*No chemo*	*7 (25%)*
2. Clinical Decision	22 (20.9%)
3. Unknown/Other	55 (52.4%)

Pending	39 (15.4%)

Clinical centres open for recruitment to SPARE were asked to keep detailed logs of patients who were investigated for trial eligibility to record what happened to them, including reasons for including/not including them in recruitment and any reasons given for declining involvement. The analysis of these data is shown in Table 2.

The qualitative analyses of the data gained during Phase I presented a clear picture of the recruitment issues. In particular, there were two key issues comprising five challenges that hindered recruitment to the SPARE trial - the first had its origins in detailed aspects of the trial design and conduct; the second involved the difficulties recruiters experienced because of their perception that patients had clear treatment preferences. These key issues are outlined below, followed by a description of the plan for Phase II, and its brief evaluation.

### Key recruitment issues in the SPARE trial

#### 1. SPARE trial design and conduct

The design of the SPARE trial was not easily described by any of the investigators or recruiters in the in-depth interviews, and interviewees and the recruitment investigation team felt the PIS contained several aspects that were potentially confusing. This made attempts to recruit participants very difficult. There were four major issues:

##### (a) Complexity of trial design

Several linked aspects of the study design needed to be described to patients, including the need for neo-adjuvant chemotherapy, the timing of randomisation in relation to the cycles of chemotherapy, and two very different treatment arms: either surgery to remove the cancer and bladder, or a policy (SBP) that involved radiotherapy to destroy the cancer and preserve the bladder except where the tumour persisted when surgery was recommended. There was also discussion of the need for repeated cycles of chemotherapy and a final cystoscopy in the SBP arm that would determine the need for surgery or not. Recruiters and investigators agreed that the SPARE trial was difficult to explain:

*P4: The other trials that I work on that are randomised are usually working say between two different types of chemotherapy (...) and no, I haven't had problems with patients going into studies like that. I think it's the fact that it is sort of the radical choice if you like, surgery-no bladder, bladder preservation-keep your bladder (Nurse, Recruiter)*.

*P5: I think one of the important things to get across to them is that we are only discussing what'll happen if they had a good response to chemotherapy, and making it quite clear that if they don't have a good response to chemotherapy, then we'd be offering cystectomy anyway, because a couple of the patients I've seen have been a bit confused about the fact that they think that regardless of what happens with the chemotherapy, they still have a choice between the two, so making it very clear that we don't know whether you're going to be in this randomisation until you've had that post-chemo cystoscopy (Oncologist, Recruiter)*.

A recorded explanation of the SPARE trial given to a patient illustrates how difficult it was to explain and follow:

*P12: You've got this sort of bladder cancer, a man of your age, they say what you should have is having the bladder removed for the cancer. Um and the chemotherapy then, you know, we would suggest you add to that. Uh and as I say, that is the gold standard way of dealing with the treatment, with that approach (...)*.

*Patient: Mmm*.

*P12: The other approach that we're testing in this trial and what we're doing is seeing whether this approach works as well as um, is seeing whether we can, instead of doing an operation, whether we can use radiotherapy to sterilise the bladder. We have used radiotherapy on and off but the concern has always been about how successful it is at actually getting rid of the cancer 100%. So what we're doing within this trial is testing whether or not, compared to having surgery, whether or not we could select people who've had chemotherapy. So if you get this part of the treatment, you would be randomised to having radiotherapy treatment if the cancer's gone after your chemotherapy compared to um, and if the cancer hasn't gone you still have surgery (...)*.

*Patient: Mmm*.

*P12: This approach we call selective bladder preservation. At the moment, we have no idea how that stacks up against surgery in terms of how good it is*.

*Patient: Mmm*.

*P12: If we could show that that approach was as good as surgery then it may be that'd be a quite an attractive treatment, but, and it may sound an attractive treatment but you know our first mission is to cure you, the second is to cure you in the best possible way we can*.

*Patient: Mmm...hmm*.

*P12: So you could certainly argue the most certain way of doing that is to do the surgery, um a less certain way might be to do radiotherapy, but you might have the advantage of not needing this big operation (Recruiter, recruiter-patient interaction)*.

The complexity of the trial design led to confusion among some recruiters about the timing of randomisation:

*P10: I explain to them that they would normally come up against randomisation if the initial cystoscopy after 2 cycles is it, 3 cycles, 2 cycles I think, was positive (Surgeon, Recruiter)*.

Recruiters indicated that they found the quantity of information problematic as well as its complexity:

*P4: I felt it really difficult and almost embarrassed at times with the amount of information, not just the SPARE information sheet which is, you know, quite wordy, quite lengthy, and then the biological samples, and then the quality of life, and the qualitative study. You just thought, 'gosh, this is awful', because they've also just been given information on gemcitabine and cisplatin and the side effects, they've just been informed that their bed's been booked for this, I mean it's a huge amount to take on, and I think that sometimes it's most probably just easier just to say 'no', than to think about it (...) it's really difficult having to sort of bombard patients very early on with so many different information sheets and consent forms (Nurse, Recruiter)*.

##### (b) Recruitment pathway

Recruiters were asked to describe the pathway that potential trial participants followed from a diagnosis of bladder cancer to being recruited to the SPARE trial. In all centres this proved extremely difficult and convoluted because of the number of people who might come into contact with the patient during their visits and sometimes the different clinical (surgery or oncology, or local/regional) centres that might be involved:

*P3: I think what we didn't appreciate was the number of the different pathways with which people actually come into that system, and the complexity (...) in terms of the treating centres and the randomising centres and all the different centres that are involved in an individual patient's care (Investigator)*.

*P1: You may have multiple different people involved in the patient's care, all of whom may give a slightly different spin on the information, and some might be very heavily involved in the trial and some people might be very peripheral to it (...) different teams do different parts of the treatment, is that some parts of the team might be more committed than other parts of team (Oncologist, Recruiter)*.

Recruiters also believed that some teams or members were very committed to SPARE but that others were indifferent or even antagonistic to it, and this created additional difficulties because patients developed strong preferences for one arm or the other:

*P1: Patients have had very fixed ideas about what treatment they want, which is I think a consequence of not having the whole team on board and that can happen at centres where they're referred in from outside, so they've spoken to a urologist about surgery before anyone even thought about SPARE. So the patient's primed for one particular treatment before they even get into the SPARE centre (Oncologist, Recruiter)*.

*P9: In my view the patient gets a bias towards surgery from the start (...) The patient tends to hang on to the first thing, so even if the patient gets to a radiotherapist (...) they quite often say, "oh yes, I'll have surgery" even if they get to the radiotherapist. By the time they get to me for what is supposed to be the pros and cons and the discussions around randomisation, they quite often have things set in their mind (Oncologist, Recruiter)*.

##### (c) Terminology

The protocol and PIS presented a clear story about the treatments offered in the SPARE trial in terms of their history:

*PIS, v.5: For many years the standard treatment for bladder cancer has been with immediate surgery removing the whole of the bladder - this is called cystectomy (...). Radiotherapy has been used as an alternative treatment to surgery. This is a new approach to the treatment of bladder cancer (...). Selective bladder preservation seems an attractive option but we do not know if it is as safe and successful as standard treatment (surgery)*.

This presentation was reflected by investigators and recruiters, but they consistently added the terms 'gold standard' and 'experimental':

*P1: I normally talk about the fact that we need to do chemotherapy ... and that the surgery is the 'gold standard' way of managing the cancer and that there is a possibility of the selective bladder preservation possibly being an alternative, but we need to confirm that in the trial and that's why we're doing a trial (Oncologist, Recruiter)*.

*P6: They're either having the 'gold standard' treatment or an experimental treatment (Nurse, Recruiter)*.

*P5: Both of these treatments outside of the trial are being used, and although the 'gold standard' is the cystectomy, I think it's a very important trial, because there's plenty of people opting for selective bladder preservation, but without comparison, we really don't know how they compare (Oncologist, Recruiter)*.

The terms 'gold standard' and 'experimental' are interesting because, as with other terms used in RCTs, they are 'loaded' with meaning [16]. Two recruiters had some insight into the potential impact of the terms:

*P7: I have taken quite a firm approach in what we would say is the accepted standard treatment and with the guidance notes that *(the TMG) *sent out to investigators, that's the approach that *(was) *suggested to investigators to take. Because originally we had a lot of patients preferring radiotherapy rather than surgery, so *(TMG) *issued the guidance documents after an investigators meeting (...) over a year ago where *(it was) *said, "it should be emphasised that the 'gold standard' treatment internationally is still cystectomy" and I think then we had a little bit of a backlash where may be we were selling it too much and so more *(laughing) *patients decided to go down the cystectomy arm (Oncologist, Recruiter)*.

*P1: Certainly in my experience *(there is an overall preference among patients for the SBP arm)*, but whether that is something that we're unconsciously telling patients and... they're reading those signs... (Oncologist, Recruiter)*.

The similarity of these phrases to other 'loaded' terms in other RCTs [16] meant that these were identified as key issues to address in a redrafted PIS and advice to recruiters in Phase II.

##### (d) Eligibility issues

As indicated above, the screening logs revealed that only small numbers of patients were deemed to meet *all *the inclusion/exclusion criteria and, even amongst those who were eligible, many were not approached about the trial, and only a very small number agreed to consider randomisation. Reasons for this included the complexity of the eligibility criteria:

*P7: Few eligible patients, well, far fewer than we originally thought and it's the eligibility for chemotherapy, fitness for chemotherapy, some patients who are not radiotherapy candidates because of specific things like, they've got a lot of non-tumor associated carcinoma-in-situ (Oncologist, Recruiter)*.

The small numbers also meant that recruiters probably forgot about SPARE:

*P2: I think there are less patients who are eligible than was originally anticipated when it was designed, because patients may be fit for surgery, but they might not be fit for chemotherapy and vice versa (...) then because of that, it makes it harder for people to may be keep it in their forefront of their minds, they might not realise that it's potential SPARE patient until too late (Investigator)*.

Some recruiters thought there was leeway for interpretation of the inclusion/exclusion criteria in partnership with the main trial team while others felt that these criteria were set in stone:

*P9: I personally don't have a problem *(with applying the eligibility criteria)*, but that's because I deal with trials all the time (...). I'm used to the fact that there's sometimes a little bit of leeway and room for interpretation and that we can always check with the principal investigator, but I think with some of my colleagues, both juniors within oncology and colleagues in surgery are not as familiar with trials, maybe have a little more difficulty in interpretation (Oncologist, Recruiter)*.

*P10: Well no, but *(inaudible) *exclusion criteria, they're kind of set in stone, aren't they. I wouldn't expect anybody, central person in the trial to encourage variance from their own exclusion criteria, would you? (Surgeon, Recruiter)*.

In summary, several aspects of the design and conduct of SPARE came together to make recruitment difficult. There was also another major difficulty - treatment preferences expressed by patients.

#### 2. Treatment preferences

Recruiters and investigators repeatedly mentioned that they were convinced that a major barrier to recruitment to SPARE was the existence of clear treatment preferences among patients:

*P1: I think I was surprised about the strength of feeling many people have about personal autonomy (...). The biggest handicap in terms of recruitment has been people saying I want one specific treatment for one particular reason or this treatment's right for me or not (Oncologist, Recruiter)*.

*P9: Either people want it *[the cancer/bladder] *out desperately, don't want to talk about anything unless it's out, they want rid of it. Or the group of patients that really doesn't pander to the idea of a big operation and want to hang on to their bladder, they want to be normal. And so I find a lot of patients come along, even if they haven't been influenced by somebody else, even if the trial has been put to them beautifully, they usually have some kind of preference based on what happens to them rather than on the effects or the efficacy of the treatment, and I think that's the biggest problem from the patient's point of view (Oncologist, Recruiter)*.

Some thought that patients' preferences were influenced by others involved in the recruitment pathway:

*P6: Depends who's spoken *(laughs lightly) *to them first, I mean if the surgeons have spoken to them first, they generally think that surgery is the best option because it's been discussed by a surgeon and to a surgeon that is the best option. If they've spoken to an oncologist or myself, if they speak to me, they tend to be more open-minded about it, because I present both sides. It's quite difficult to get people onto a trial saying, 'hey lets see if it's alright to not have surgery', when the surgeons are saying, 'well I'd have surgery'. It's not that *(surgeon) *is anti-trial or anything else, because he's not... he's very pro trials, but his fundamental belief is that if you've got a bladder cancer you should have your bladder taken out (Nurse, Recruiter)*.

*P2: Some people, urologists especially, in some centres are not behind the trial at all, and will not put patients in towards it, so some centres I know haven't recruited because there's a problem with the urologist. Clinicians in general are doing what they think is best, so surgeons are very defensive of cystectomy because they've done lots of them, and they think that that's the best thing to do, and some of them haven't accepted that the best thing to do is put patients into SPARE (Investigator)*.

Most recruiters felt they should accept any expressed preferences, and were reluctant to challenge patients' preferences:

*P6: *(Challenging patient's preferences) *depends very much on the patient. If I feel that they're the sort of person who could cope with the uncertainty, then I'll say to them, 'we're not going to know unless research is done' or say to them, 'it wouldn't be being looked at if there wasn't very good evidence showing that radiotherapy was potentially as good', but you can tell when you're speaking to patients, if they absolutely want the bladder removed, you're not going to be able to sway them, and I don't think it would be ethical to try to be honest. If they're adamant, no they want it over with, they want it out, they want it gone, then I don't think it's ethical to push, because if it wasn't the trial, they wouldn't be having radiotherapy pushed on them anyway, and I don't feel it's ethical to push them any further (Nurse, Recruiter)*.

Most recruiters did indicate their own treatment preferences and most also insisted that they did not transfer these onto patients:

*P5: I quite like the idea of selective bladder preservation with radiotherapy, it doesn't mean I sell that over and above everything else to my patients (Oncologist, Recruiter)*.

*P6: If it was me *(sighs) *yeah, I'd probably want it taken out, but I can be very positive *(laughs) *when I need to be, I'm very pro research (...) I don't think my preferences would affect what I say because I keep that inside my head. I'm very good at, you know, at the end of the day my job is to promote research, and I think it's a question that needs answering and I truly don't think that my personal views would even come into it (Nurse, Recruiter)*.

Later, this same recruiter continued:

*P6: I think they've got to be pro radiotherapy to be able to go in the trial. I certainly wouldn't try and influence someone at all (Nurse, Recruiter)*.

It is clear from other research that the personal views of recruiters can be transmitted to patients, often unwittingly [17]. In the following extract, this same recruiter continues to convey their personal views and also shows a willingness to act on judgements about patients that might explain why less than half of patients eligible for SPARE had the trial explained to them:

*P6: I think if you've got a very anxious, nervous patient, they're not going to go on the study anyway, because they'd rather just have it taken out and be done with it. I think you've got to have quite a confident person to be on a trial like this. I can't say to someone, 'I'm going to give you gold standard treatment and we're going to look at extra', they're either having the gold standard treatment or an experimental treatment. You're asking someone to give it a try, and whilst I think that's great, and we need to do it, it's a lot for someone to take on board (Nurse, Recruiter)*.

A further factor that emerged from the interviews was that centres sometimes appeared to take on a 'collective' preference - one that represented the views of most staff in the centre. Two out of the four centres interviewed gravitated to a collective view - one for surgery and the other for SBP. The screening logs from these two centres also reflected the collective preference of the centre. In the centre that appeared to be pro-SBP, 7 out of 9 patients who declined did so because of a preference for the bladder preservation arm or because they did not want surgery. Similarly, in the centre that appeared to be pro-surgery, 4 out of 6 patients who declined opted for surgery.

### Plan of action - Phase II

An overview of the findings above was presented to the SPARE TMG and the following actions were agreed:

#### (a) Feasibility study to continue

It was agreed that the recently published review article [5] gave new impetus to the need for the SPARE trial. Interview data showed that investigators and recruiters, particularly based in oncology, continued to view this as an important question, and that there was strong commitment from clinicians and recruiters.

#### (b) Changes to design and paperwork

**Figure 1:**
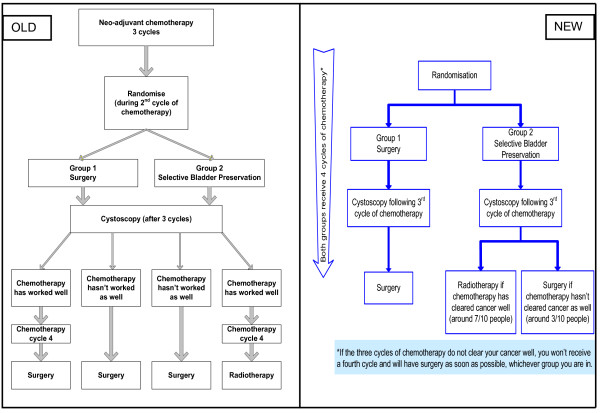
**SPARE trial treatment scheme flowchart in Patient Information Sheet: Old and New**. Following findings from the qualitative recruitment investigation that the SPARE trial was difficult to explain, a simpler version of the study flowchart was incorporated in all study documentation including the Patient Information Sheet (PIS). The old and new versions of the study flowchart in the PIS are provided in Figure 1.

It was accepted that the trial design was too complex to be easily articulated and that some of the terminology in the PIS was potentially confusing. Intensive discussion of the intended design led to the construction of a simpler version of the study flowchart (Figure 1) which was then issued to recruiters so that they could provide a clearer articulation of the trial. The consent for chemotherapy was separated from the consent for SPARE in response to recruiters indicating that patients were given too much information about various aspects of the trial at the same time. The randomisation period was also simplified and clarified so that patients could be randomised at any time before the three cycles of chemotherapy rather than during the second cycle.

The recruitment study team drafted a new, shorter and clearer PIS which removed the 'loaded' terminology, explained the simplified study outline and included the new flowchart. The PIS also included a clearer explanation of randomisation and a new table comparing evidence about side effects and outcomes of each treatment. These changes drew on findings from the interviews and the content of PIS' from other RCTs.

#### (c) Recruitment pathway streamlining

**Figure 2:**
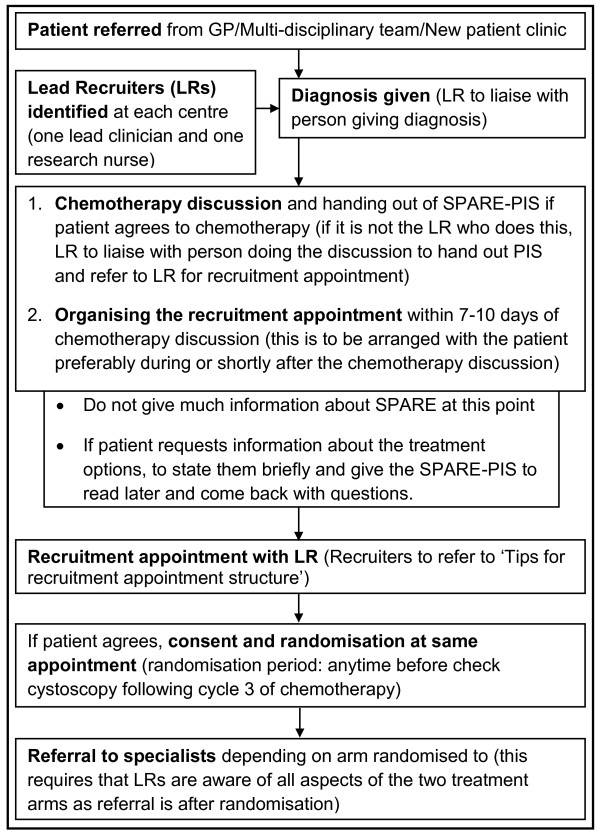
**Recommendations to streamline the SPARE trial recruitment pathway**. Following findings from the qualitative recruitment investigation that the SPARE trial recruitment pathway was complicated and involved a number of people, suggestions to streamline the recruitment pathway were provided to recruitment centres. The schematic representation of the suggestions as distributed to centres is shown in Figure 2.

It was agreed that the involvement of many different clinicians and researchers during the sensitive recruitment period was not helpful to the trial. Clinical centres were asked to identify two Lead Recruiters (LRs) per site whose responsibilities would be to act as the focus for SPARE recruitment activity. The LRs were also advised to see if they could arrange a specific 'recruitment appointment' about 7-10 days after the chemotherapy discussion, with the aim of providing full information about the trial and treatments, and obtaining consent for participation and randomisation if possible. It was also recommended that trial participants should be referred to the respective specialists after randomisation rather than before to ensure consistency of information and to avoid information overload (Figure 2).

#### (d) Eligibility

Problems with finding sufficient numbers of eligible patients emerged as a critical issue, especially during the training and feedback process:

*P9: It's funny we just seem to keep finding extra ways for patients not to be eligible. It's quite bizarre really and it's gone beyond despair and got to be a bit of a joke in a way, every time we think we've got a good candidate, there's something going on *(laughs) *(Oncologist, Recruiter)*.

*P15: When we went back and had a look at it, the consultant came back towards the surgeon and said, 'oh there isn't as many as I originally thought', and sort of admitted that perhaps he'd overestimated the amount of patients to when he'd gone back and had another look, so I don't think we, they just don't come through that regularly (training teleconference)*.

The possibility of relaxing certain inclusion criteria was discussed with the TMG but it was decided that these could not be changed without invalidating the aims of the RCT.

#### (e) Training and individual feedback

In order to address the issues related to treatment preferences and to ensure that the new SPARE trial information was acceptable and used by recruiters, several training sessions were organised. The first two were held at conferences (British Uro-oncology Group Annual Meeting and National Cancer Research Institute Conference). The content included a summary of the findings from Phase I, the changes suggested to study documentation, and some simple 'tips' about recruitment drawn from the ProtecT documents [10]. There were then four recruitment training sessions with particular centres (two by teleconference and two face-to-face). These sessions included examples of good practice and recruitment 'tips' included those that were transferable from the ProtecT study and some developed specifically for the SPARE trial. Transferable tips included the need to let patients know that they were eligible for both treatment arms and that the evidence for which treatment was better was still unknown [16], systematically eliciting and addressing patient preferences/concerns, and the use of strategies such as readily ceding the floor when overlapping speech occurs to enable patients to express concerns that may otherwise be concealed [17]. Tips specific to the SPARE study included providing information about chemotherapy separately from the radiotherapy/surgery treatment options, the practicalities of implementing the streamlining of the recruitment pathway, and options for addressing patient preferences using case studies provided by SPARE recruiters. Individual feedback on the content of audio-taped recruitment encounters was given to the recruiters who provided tape-recordings.

### Outcome of Phase II

A table showing the major findings and the subsequent changes and methods of implementation provides an overview of the potential impact of the recruitment study (Table 3). The extremely tight timelines for the study meant that evaluating the impact of the changes made in Phase II was difficult. At the request of the Independent Data Monitoring Committee (IDMC) a joint meeting of the IDMC and TSC was held in January 2010 to review recruitment in light of the target of six patients per month ahead of the submission of the annual report to the funding body. Although informed of the alterations to SPARE and the training implemented as a result of this study, with average recruitment of one patient per month, the TSC considered that the issues related to restricted patient eligibility were insurmountable and advised closure of the SPARE trial in February 2010. As this was only six weeks after the last training session and included the winter holiday period, there were only very limited data that could indicate whether the changes made might have had an impact.

**Table 3:** SPARE trial qualitative recruitment investigation - mapping findings and changes

Key issues	Findings	Changes/recommendations	Aspect of trial changed/recommendation for	Implementation
		Simplified flowchart	PIS and protocol	^a ^Amended and ethical approval obtained
	Difficulty in explaining the trial			
		Easy explanation of trial	Recruiter-patient interaction	^b ^Training session
	
	Randomisation period confusion	Opened up randomisation period	Protocol	^a ^Amended and ethical approval obtained
	
**Aspects of trial design and conduct**	Information overload	Two-stage information provision (chemo and post-chemo)	Recruitment and pre-recruitment interactions (also linked to streamlining of pathway)	^b ^Training session ^b ^Recruitment appointment tips document
	
	Complex recruitment pathway	Streamlining of recruitment pathway and information provision	Recruitment pathway	^b ^Streamlining recommendations document
	
			PIS	^a ^Amended and ethical approval obtained
	Loaded terminology and unbalanced information	Use of neutral terms and equivalent information on both arms		
			Recruiter-patient interaction	^b ^Training session ^b ^Recruitment appointment tips document

	Patients' strong treatment preferences	Eliciting and addressing concerns/preferences	Recruiter-patient interaction	^b ^Training session ^b ^Recruitment appointment tips document
		
**Preferences**	Patient preferences influenced by recruiter, and number of people in recruitment pathway	Acknowledging and reflecting on own preferences;	Recruiter-patient interaction	^b ^Training session
		Streamlining and limiting number of people involved in recruitment pathway	Recruitment pathway	^b ^Streamlining recommendations document

^a ^these changes of the PIS and protocol were done together

^b ^these aspects were done at various points in the study, but it was not possible to monitor their implementation because of the closure of the trial

The major findings and the subsequent changes and methods of implementation of the qualitative recruitment investigation are shown in Table 3.

Feedback was sought about the various changes and tips during the training sessions and there was overall agreement that these had been helpful:

*P13: The change in when you can randomise patients, that's made a big difference, having that little bit more playing time (training teleconference)*.

*P18: Well I noticed that it *(flowchart) *was much simplified, simplified, and much easier, yes (training teleconference)*.

*P9: I quite like the idea of bringing randomisation further forward, I think it's in an odd place at the moment (...). I like the flow sheet, figure *1, *diagram of SPARE treatment is a great improvement. I believe the patients on the previous one that had the four way tracks down as it were, I think that's a big help (...). In the latest version of the information sheet, which I think is version 6 with that lovely table on the end which has the pros and cons, if we gave that to patients before a surgeon spoke to them, before a radiotherapist spoke to them, heaven's sake, before a medical oncologist spoke to them, then we might have some patients who better appreciated the difficulties involved (Oncologist, Recruiter)*.

Most recruiters felt that the recommendations to streamline the recruitment pathway were sensible and feasible in their centres:

*P9: I like the idea fundamentally, it fits in with a lot of things, a lot of conclusions I've been coming to (Oncologist, Recruiter)*.

*P10: I can see the reasoning behind that yeah. I can't see that being a problem here no (Surgeon, Recruiter)*.

The main focus of the training sessions was to enable recruiters to manage treatment preferences and there was some evidence that it helped recruiters feel more confident in this area:

*JLD: Do you feel comfortable doing that *(addressing preferences)*?*

*P17: I haven't done previously, but I think after speaking to you today*, (you) *expressing that it is a common problem and that they might not be fully informed about it, I think I'll probably feel a bit more confident in approaching it and discussing both options and enlightening them on both options (training teleconference)*.

In terms of numbers of new recruits, there was insufficient time after the training sessions to be conclusive. Two centres, one that had recruited only two patients over 25 months and the other one patient over 6 months, expressed renewed enthusiasm after attending the training sessions and then had discussions with three eligible patients, one of whom agreed to randomisation shortly before the closure of the trial.

## Discussion

Qualitative research methods were used to investigate reasons for low levels of recruitment to the SPARE trial which compared two very different treatments for bladder cancer: surgical removal of the bladder versus a policy of radiotherapy to preserve the bladder in most cases. Within three months, a combination of documentary analysis, in-depth interviews with investigators and recruiters, simple analysis of eligibility/screening logs, and tape-recording of a small number of recruitment interactions clearly identified four major barriers to recruitment in relation to the design and conduct of the trial, and another major obstacle in the form of strongly expressed patient treatment preferences. A plan to improve recruitment was drawn up and various changes were implemented (new PIS and study flowchart, streamlining of the recruitment pathway), followed by dedicated training sessions and information to disseminate the changes and tackle the issue of treatment preferences. One issue was intractable - the shortage of eligible patients - and this contributed to the TSC's decision to close the trial (on the grounds that the large phase III trial, powered for non-inferiority of SBP, would not be feasible) [18]. Several comments were made by recruiters and investigators that the changes were plausible and the training sessions acceptable and useful, and there was also an indication in two centres that renewed enthusiasm had led to new ways of working and might have been able to generate new trial recruits, but a clear evaluation of the outcome of the plan to improve recruitment could not be undertaken. The scale of the recruitment target and the lack of eligible patients proved fatal to the SPARE RCT.

This recruitment investigation built on considerable previous research, particularly the development of a complex recruitment intervention based on the ProtecT study [10] which was then applied to five different RCTs in the Quartet study (Qualitative Research to Improve Recruitment to Randomised Controlled Trials) [7]. The SPARE study has enabled further refinement of the intervention. Several problems that arose in these previous studies were avoided in the SPARE study by, for example, ensuring ethical approval for the qualitative work was integrated into the trial, interviews were conducted as rapidly as possible, documentary analysis was undertaken to investigate the evidence for the trial still being an important question and the PIS was scrutinised to ensure it provided a simple and balanced view of the treatment options and a clear explanation of randomisation.

Some of the issues identified early in the recruitment investigation required changes that were fairly unproblematic to implement. For instance, the finding that the complexity of the trial design was proving to be a major obstacle to recruitment provided an ideal opportunity to discuss the clear questions being addressed at the heart of the trial that could then be captured in a new flowchart and PIS. Essentially, SPARE was a comparison of surgical removal of the bladder versus an attempt to retain the bladder. The complex issues of chemotherapy, timing of cystoscopy and also the final determination of whether surgery would be necessary in the SBP arm could then be more easily described within that framework.

The discovery of unexpectedly 'loaded' terminology in the interviews and recordings of appointments was similar to the ProtecT study, although the actual 'loaded' terms were different. This shows the importance of understanding how common medical terms are perceived in particular circumstances, and how their avoidance might improve recruitment [16]. In the SPARE trial, it was important to convey that cystectomy was the long-established treatment, and that the policy of radiotherapy to allow selective bladder preservation was a newer concept that potentially offered a less invasive treatment - and that a comparison of the two approaches was urgently required. This became translated to surgery being the 'gold standard' and SBP 'experimental' and thus led to the reinforcement of treatment preferences that were already strong because of the differences perceived between the arms [6]. Interestingly, the 'loaded' terms could be detrimental or supportive, depending on the direction of the underlying preference.

The emergence of patients' treatment preferences as a major barrier to recruitment was not unexpected because of the similarity to the surgery/radiotherapy comparison in the ProtecT study and in two of the Quartet trials [7]. These preferences were also identified, to some extent, through screening logs collected by the trial team [19] and in the qualitative patient interview study [6]. Patients' treatment preferences have been identified as a barrier to recruitment in many systematic reviews [20-23]. However, in the ProtecT study complex recruitment intervention, training was given to enable recruiters to learn how to elicit, understand and then, where appropriate, address patient preferences [17], leading to a clear increase in randomisation and informed consent [16]. There were examples in the ProtecT study of recruiters unwittingly passing on their own biases to patients [16], and this was also found in the SPARE trial, but another concept also emerged here - that of the development of a shared 'centre preference'. Within particular centres, members of the clinical team had preferences themselves for a particular treatment, and then (mostly unwittingly) transmitted these to patients. This was evident in interviews and was confirmed by screening logs in which patients in particular centres tended to decline participation because of a preference for one arm or the other. The development of different 'centre' preferences is not unexpected when there is uncertainty over treatments - so called 'community equipoise' [24,25] - but unless this is tackled it is problematic for trial recruitment.

The SPARE training sessions drew on generic recruitment tips from the ProtecT and Quartet study findings. The development of specific tips for SPARE was hindered by the failure to collect audio recordings of appointments - only four were available. The reasons for the failure of recruiters to record themselves despite indicating a willingness to do so were not specifically elicited in this study, but are likely to be similar to those found elsewhere, including recruiters feeling daunted by the prospect of being scrutinised/criticised, and fearing that their role as the patient's advocate might be compromised by having to ask about the recording [7]. As this is such a crucial aspect for understanding recruitment interactions, there needs to be more research in future to counter these fears and increase the number of recordings.

An important contribution of the SPARE recruitment study was to show that it was possible to apply a rapid adaptation of a ProtecT-like recruitment intervention, using primarily qualitative research methods. The key issues were identified within three months, a plan of action agreed in month four and implemented in months five to seven.

Screening logs provided helpful information about the application of eligibility criteria and the number of potential participants. The SPARE trial conformed with all the Good Clinical Practice (GCP) criteria and was extremely well co-ordinated. The TMG was aware that recruitment was difficult, but it was the targeted qualitative research that elicited the underlying barriers to recruitment, including the difficulties recruiters experienced in explaining the design simply and the interactions between clinical teams in centres that created 'centre preferences'.

The SPARE recruitment study has identified a number of issues relevant for the future design of trials. It is important, for example, to reduce the apparent complexity of the trial by encapsulating the design in a relatively simple flowchart. This then helps recruiters to be clearer in their presentation of the trial. The SPARE study also indicated several issues relevant to the future application of recruitment interventions that incorporate qualitative research. Although the recruitment investigation and its intervention were completed and implemented quickly, the trial closed before its findings could be fully evaluated, so the optimal time for such studies might be early in the trial design or feasibility stage - as in the ProtecT study [16]. Qualitative research that is initiated at later stages faces a number of challenges [7]. However, the time-lines for the intervention are now clearer from this study, and so targeted investigations might be possible if there were at least 18 (or better 24) months of recruitment time remaining. Another factor of importance for the future for trials with very different arms is to explore the need for joint lead investigators representing each clinical speciality. The intervention part of the study would have had much more and earlier immediate relevance for SPARE if it had been possible to obtain audio-recordings of recruiter-patient interactions. This aspect should perhaps have greater prominence in future interventions. The success of the qualitative investigation in understanding the key recruitment issues inside three months bodes well for future applications. It is likely that these sorts of studies are more useful for trials that involve very different treatment arms - as in ProtecT and SPARE. This was also true of the Quartet trials, although several of those were 'difficult' for other reasons [7,8]. Such interventions are unlikely to be necessary in placebo-controlled drug trials, or those involving different methods of delivery of the same treatment. There are, however, increasing numbers of complex and challenging trials that may provide opportunities for targeted recruitment interventions.

## Conclusions

In conclusion, this study has demonstrated the adaptability of the ProtecT intervention and contributed towards refining the methods for future recruitment interventions using qualitative research methods. It has underlined the role of such studies in elucidating challenges to recruitment in trials with very different treatment arms, and has given some hope that these challenges may not be insurmountable if the time required for the intervention to take effect is provided.

## List of abbreviations

CRUK: Cancer Research UK; GCP: Good Clinical Practice; ICR-CTSU: Clinical Trials & Statistics Unit; IDMC: Independent Data Monitoring Committee; ISRCTN: International Standard Randomised Controlled Trial Number; LR: Lead Recruiter; NIHR: National Institute for Health Research; PIS: Patient Information Sheet; ProtecT: Prostate testing for cancer and Treatment; Quartet: Qualitative research to improve recruitment to randomised controlled trials; RCT: Randomised Controlled Trial; SBP: Selective Bladder Preservation; SPARE: Selective bladder Preservation Against Radical Excision; TMG: Trial Management Group; TSC: Trial Steering Committee.

## Competing interests

The authors declare that they have no competing interests.

## Authors' contributions

JLD designed and led the study, contributed to the data analysis and led the redrafting of the manuscript. SP carried out the data collection and analysis and produced the first draft of the manuscript. EH, RL, AB and RH contributed to the study conception and design and contributed to the redrafting of the manuscript. All authors read and approved the final manuscript.

## Authors' information

JLD was the Chief Investigator of the recruitment study and SP was employed as a Research Assistant on the study. RH was the Chief Investigator of the SPARE trial and AB was the Clinical Co-ordinator. EH was the Trial Statistician and responsible for the management of the trial at ICR-CTSU. RL was the Trial Manager for the SPARE trial.

## References

[B1] Britton A McKee M Black N McPherson K Sanderson C Bain C Choosing between randomised and non-randomised studies: a systematic review Health Technol Assess 1998 2 1 124 9793791

[B2] Ergina PL Cook JA Blazeby JA Boutron I Clavein P Reeves BC Seiler CM for the Balliol Collaboration Surgical innovation and evaluation 2: Challenges in evaluating surgical innovation Lancet 2009 374 1097 1104 10.1016/S0140-6736(09)61086-2 19782875 PMC2855679

[B3] McCulloch P Altman DG Campbell WB Flum DR Glasziou P Marshall JC Nicholl J for the Balliol Collaboration Surgical innovation and evaluation 3: No surgical innovation without evaluation: the IDEAL recommendations Lancet 2009 374 1105 1112 10.1016/S0140-6736(09)61116-8 19782876

[B4] Reeves B Health-technology assessment in surgery Lancet 1999 353 3 5 10.1016/S0140-6736(99)90413-0 10319922

[B5] Kaufman DS Shipley WU Feldman AS Bladder cancer Lancet 2009 374 239 249 10.1016/S0140-6736(09)60491-8 19520422

[B6] Moynihan C Hall E Lewis R Birtle A Mead GM Sreenivasan T Huddart R on behalf of the SPARE TMG SPARE: A qualitative study investigating randomisation barriers in a Selective Bladder Preservation trial (SBP) (ISCRCTN:61126465) [abstract] J Clin Oncol 2009 27 s15 10.1200/JCO.2008.16.1901

[B7] de Salis I Tomlin Z Toerien M Donovan J Using qualitative research methods to improve recruitment to randomized controlled trials: the Quartet study J Health Serv Res Policy 2008 13 92 96 10.1258/jhsrp.2008.008028 18806198

[B8] de Salis I Tomlin Z Toerien M Donovan J Qualitative research to improve RCT recruitment: Issues arising in establishing research collaborations Contemp Clin Trials 2008 29 663 670 10.1016/j.cct.2008.03.003 18479977

[B9] Howard L de Salis I Tomlin Z Thornicroft G Donovan J Why is recruitment to trials difficult? An investigation into recruitment difficulties in an RCT of supported employment in patients with severe mental illness Contemp Clin Trials 2009 30 40 46 10.1016/j.cct.2008.07.007 18718555 PMC2626649

[B10] Donovan JL Lane JA Peters TJ Brindle L Salter E Gillatt D Powell P Bollina P Neal DE Hamdy FC for the ProctecT Study Group Development of a complex intervention improved randomization and informed consent in a randomized controlled trial J Clin Epidemiol 2009 62 29 36 10.1016/j.jclinepi.2008.02.010 18619811

[B11] Gomm R Social research methodology - a critical introduction 2004 Basingstoke: Palgrave Macmillan

[B12] Neuman WL Social research methods - qualitative and quantitative approaches 2001 4 Needham Heights: Allyn & Bacon

[B13] Silverman D Interpreting qualitative data - methods for analysing talk, text and interaction 2001 2 London: Sage

[B14] Donovan J Sanders C Bowling A, Ebrahim S Key issues in the analysis of qualitative data in health services research Handbook of health research methods 2005 Maidenhead: Open University Press 515 532

[B15] Miles M Huberman M Qualitative data analysis 1994 London: Sage

[B16] Donovan J Mills N Smith M Brindle L Jacoby A Peters T Frankel S Neal D Hamdy F for the ProtecT Study Group Improving design and conduct of randomised controlled trials by embedding them in qualitative research: ProtecT (prostate testing for cancer and treatment) study BMJ 2002 325 766 770 10.1136/bmj.325.7367.766 12364308 PMC1124277

[B17] Wade J Donovan JL Lane JA Neal DE Hamdy FC It's not just what you say, it's also how you say it: Opening the 'black box' of informed consent appointments in randomised controlled trials Soc Sci Med 2009 68 2018 2028 10.1016/j.socscimed.2009.02.023 19364625

[B18] Huddart RA Hall E Lewis R Birtle A Life and death of SPARE (Selective bladder Preservation Against Radical Excision): reflections on why the SPARE trial closed BJU Int 2010 106 753 755 10.1111/j.1464-410X.2010.09537.x 20707796

[B19] Lewis R Huddart R Birtle A Blazeby J Jones E Hall E on behalf of the SPARE TMG Challenges of identifying cancer patients for randomised controlled trials from urology clinics - what lessons can we learn from SPARE? (CRUK/07/011) [abstract] National Cancer Research Institute Conference 2009 Birmingham http://www.ncri.org.uk/ncriconference/2009abstracts/abstracts/C118.htm

[B20] Fayter D McDaid C Eastwood A A systematic review highlights threats to validity in studies of barriers to cancer trial participation J Clin Epidemiol 2007 60 990 1001 10.1016/j.jclinepi.2006.12.013 17884592

[B21] King M Nazareth I Lampe F Bower P Chandler M Morou M Sibbald B Lai R Impact of participant and physician intervention preferences on randomized trials: a systematic review JAMA 2005 293 1089 1099 10.1001/jama.293.9.1089 15741531

[B22] Mills EJ Seely D Rachlis B Griffith L Wu P Wilson K Ellis P Wright JR Barriers to participation in clinical trials of cancer: a meta-analysis and systematic review of patient-reported factors Lancet Oncol 2006 7 141 148 10.1016/S1470-2045(06)70576-9 16455478

[B23] Ross S Grant A Counsell C Gillespie W Russell I Prescott R Barriers to participation in randomised controlled trials: a systematic review J Clin Epidemiol 1999 52 1143 1156 10.1016/S0895-4356(99)00141-9 10580777

[B24] Gifford F Community equipoise and the ethics of randomized clinical trials Bioethics 1995 9 127 148 10.1111/j.1467-8519.1995.tb00306.x 11653056

[B25] Gifford F Uncertainty about clinical equipoise BMJ 2001 322 795 10.1136/bmj.322.7289.795 11282877 PMC1119966

